# Fertility-sparing management of atypical polypoid adenomyoma in a premenopausal woman: a two-case report and literature review

**DOI:** 10.1016/j.xagr.2025.100589

**Published:** 2025-11-27

**Authors:** Ivo Vukasović, Dinka Pavičić Baldani, Lana Škrgatić, Magdalena Karadža

**Affiliations:** 1 Department of Gynaecology and Obstetrics, University Hospital Centre Zagreb, Zagreb, Croatia (Vukasović, Baldani, Škrgatić, and Karadža); 2 School of Medicine, University of Zagreb, Zagreb, Croatia (Baldani, Škrgatić, and Karadža)

**Keywords:** atypical polypoid adenomyoma, fertility-sparing treatment, hysteroscopy

## Abstract

**Objective:**

To describe fertility-sparing management of atypical polypoid adenomyoma (APA) in two premenopausal women and to review the relevant literature on treatment strategies aimed at preserving fertility.

**Case Report:**

A 39-year-old nulliparous woman presented with heavy menstrual bleeding and was diagnosed with APA via hysteroscopy and histopathology. Rapid recurrence prompted a second-look hysteroscopy and complete lesion resection. The second case involved a 30-year-old nulliparous woman with prolonged bleeding and a suspected submucosal fibroid; hysteroscopic resection confirmed APA. She received a levonorgestrel-releasing intrauterine device and regular follow-up. Both cases highlight diagnostic and therapeutic challenges in managing APA while preserving fertility.

**Conclusion:**

These cases illustrate the diagnostic and therapeutic challenges of APA while preserving fertility. Based on our cases and a review of the literature, complete hysteroscopic resection, combined with hormonal therapy when appropriate, may be effective. Close follow-up is essential due to the risk of recurrence and potential malignant transformation, and individualized management is recommended to optimize fertility outcomes.

## Introduction

Atypical polypoid adenomyoma (APA), also known as atypical polypoid adenomyofibroma of the uterus, is a rare endometrial lesion first described by Mazur in 1981. It is characterized by atypical glands enmeshed in smooth muscle, which can mimic endometrial adenocarcinoma or a malignant mixed Müllerian tumor.^1^ The highest incidence is observed in nulliparous women of premenopausal age, which presents a significant challenge in fertility preservation. Due to the potential for recurrence and progression, fertility-sparing management is based on hysteroscopic resection with or without progestin therapy.^2^^,^^3^ In women who have completed their reproductive years, hysterectomy represents the definitive treatment option. It most commonly occurs in the fundal region of the uterus (55.8%), with abnormal uterine bleeding being the most frequent symptom.^3^ The pathophysiology remains unclear, and the risk factors for the development of APA are similar to those for endometrial carcinoma. Wang et al^4^ suggest that an elevated HOMA-IR value (>2.2) and a low HDL concentration (<1.2 mmol/L) are independent risk factors for the development of endometrial atypical hyperplasia and endometrioid endometrial cancer in women with APA, which is why these patients require rigorous clinical monitoring. Also, the high rate of complete and partial responses to high-dose oral medroxyprogesterone acetate therapy supports the hypothesis that APA is a hormone-related disease.^5^

## Case report

We present two case reports. The first case involves a 39-year-old woman who reported prolonged and heavy menstrual bleeding, which had resulted in secondary anemia. Her family history is notable for breast cancer in her maternal grandmother and colon cancer in her grandfather. She is otherwise healthy, nulliparous, BMI 21,27 kg/m^2^, and had used oral hormonal contraceptives during her mid-20s for contraception. A routine transvaginal ultrasound revealed a 10 mm intrauterine lesion. Given her symptoms and imaging findings, an operative hysteroscopy was performed. Histopathological examination of the resected tissue revealed a polypoid lesion composed in part of normal endometrial stroma and glands, and in part of smooth muscle stroma and glands lined by endometrial epithelium. Areas of epithelial atypia and squamous morules were identified. Immunohistochemical staining was negative for CD10 and caldesmon.

**Figure 1:**
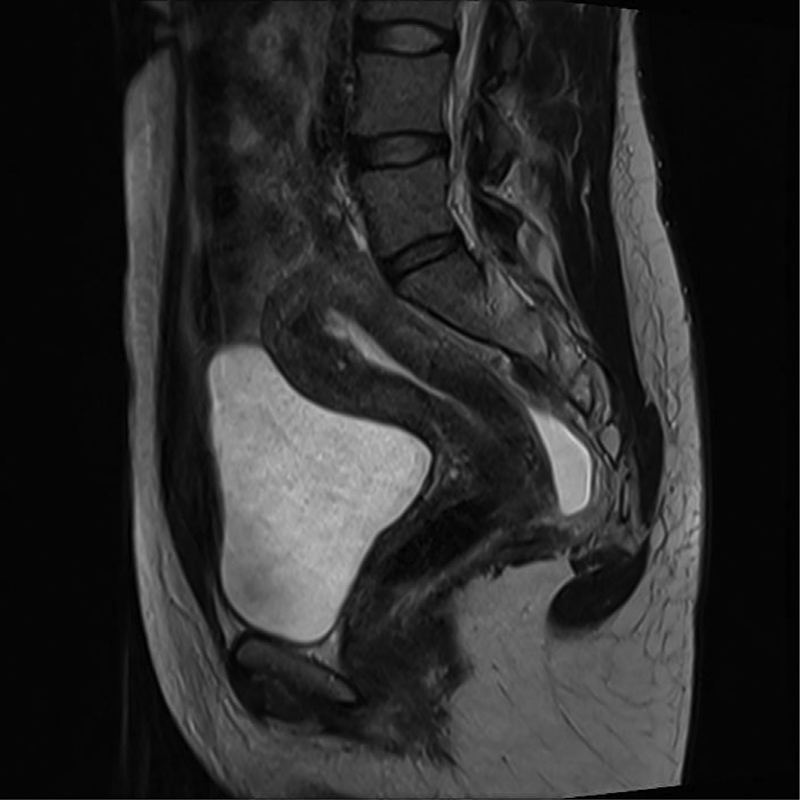
Case 1—Postoperative MRI of the anterior uterine wall showing isodense thickening

Three months later, postoperative ultrasound demonstrated a 20×18 mm intrauterine lesion located on the anterior uterine wall near the fundus, with heterogeneous echogenicity and a central hyperechoic focus measuring 9×9 mm. There was no vascular signal on color Doppler. The remainder of the pelvic ultrasound, including the myometrium and adnexa, was unremarkable. Postoperative MRI described a transitional zone on the anterior uterine wall as an isodense thickening up to 1 cm, mildly protruding into the endometrial cavity (Figure 1). There was no diffusion restriction on DWI/ADC sequences, nor any pathological contrast enhancement. Differential diagnosis included a residual lesion and scar tissue.

Vukasović et al. Fertility-sparing management of atypical polypoid adenomyoma in a premenopausal woman. AJOG Glob Rep 2025.

**Figure 2:**
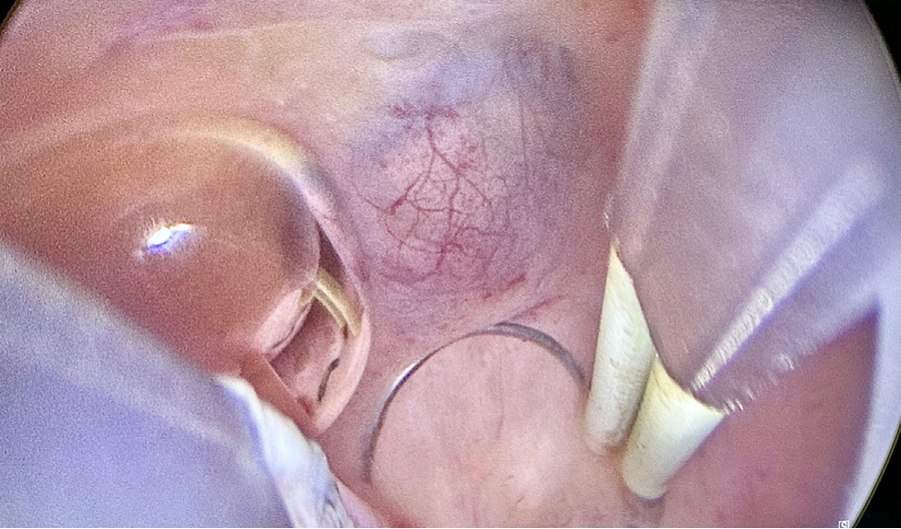
Case 1—Hysteroscopic view of a diffusely infiltrative and cystic lesion on the anterior uterine wall

In light of these findings, a second-look hysteroscopy was conducted. During hysteroscopy, a lesion was visualized on the anterior uterine wall with diffuse infiltration and cystic changes, extending from the area near the right tubal ostium across the anterior central wall and toward the fundal region, adjacent to the left lateral wall (Figure 2). Using the four-step technique described by Di Spiezio Sardo et al, the entire lesion was resected, including the surrounding endometrium and underlying myometrial tissue. Additional endometrial biopsy samples were also collected. The final histopathological analysis revealed the presence of endometrial glands and stroma within the myometrium, consistent with adenomyosis.

Vukasović et al. Fertility-sparing management of atypical polypoid adenomyoma in a premenopausal woman. AJOG Glob Rep 2025.

**Figure 3:**
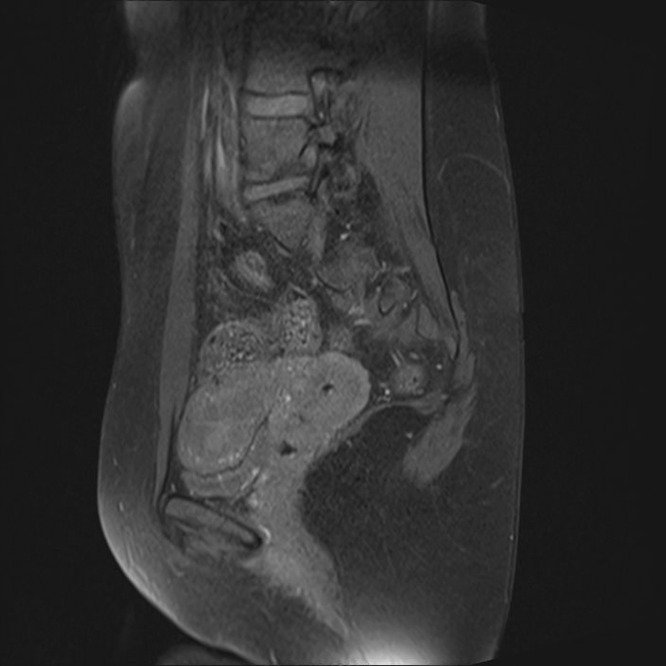
Case 2—Postoperative MRI showing a small linear contrast enhancement at the excision site, unlikely representing residual disease

**Figure 4:**
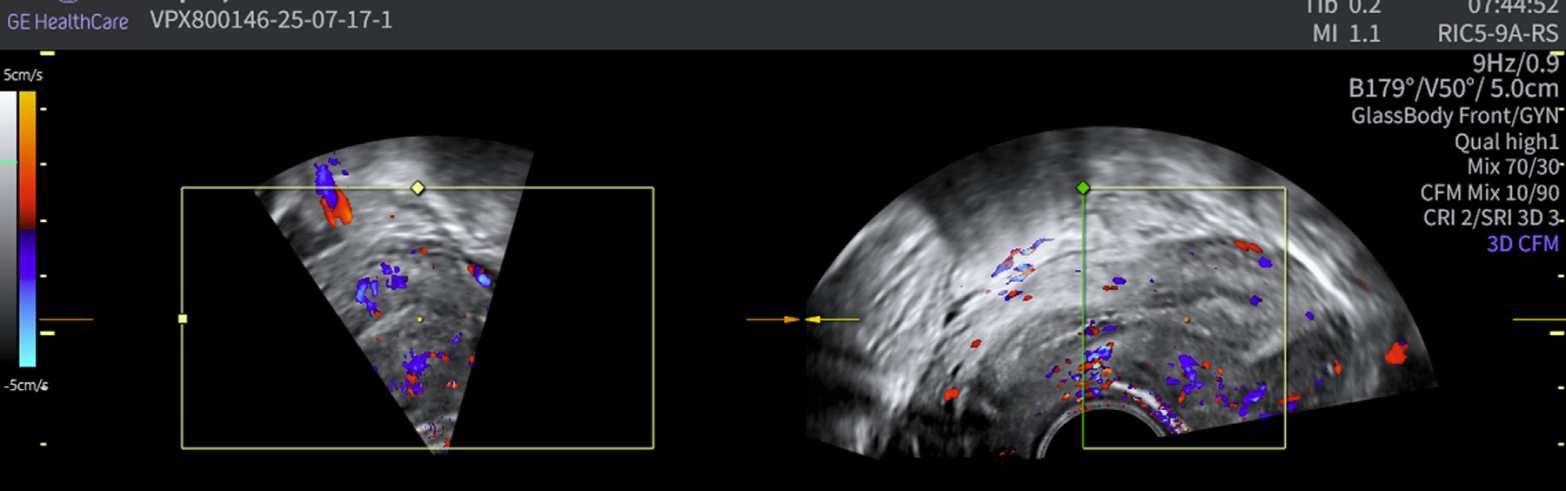
Case 2—Ultrasound 3 months after surgical procedure: regular endometrium and myometrium, clear endomyometrial junction, with no visible changes at the resection site

The second case involves a 30-year-old nulliparous woman (virgo intacta), who was referred to our clinic due to irregular and prolonged menstrual bleeding accompanied by intermenstrual spotting lasting for the past 3 months. She had a history of kidney surgery for nephrolithiasis, but was otherwise healthy. Transvaginal ultrasound revealed a hyperechogenic structure measuring 12×14 mm, suggestive of a submucosal myoma, FIGO type 1. Due to persistent bleeding unresponsive to medical therapy (dydrogesterone 10 mg orally twice daily for 14 days), hysteroscopy was performed. During hysteroscopy, a whitish lesion measuring 20×5 mm was resected on the anterior wall of the uterine cavity, near the fundus. Histopathological analysis revealed a lesion composed of smooth muscle stroma surrounded by endometrial epithelium with focal atypia and squamous morules. Immunohistochemistry showed caldesmon positivity. Three months after the procedure, MRI showed a discreet linear area of contrast enhancement at the site of the lesion excision, measuring 5 mm in length and 1 mm in thickness, visible only in the sagittal T1-weighted images (Figure 3). However, this finding is considered unlikely to represent residual disease. Figure 4 demonstrates a normal control ultrasound performed 3 months postprocedure.

Vukasović et al. Fertility-sparing management of atypical polypoid adenomyoma in a premenopausal woman. AJOG Glob Rep 2025.

Vukasović et al. Fertility-sparing management of atypical polypoid adenomyoma in a premenopausal woman. AJOG Glob Rep 2025.

The first patient was referred to a fertility clinic for IVF due to a low ovarian reserve. The second patient was offered placement of a levonorgestrel-releasing intrauterine device, along with a second-look hysteroscopy, to preserve the uterus and maintain fertility. Regular follow-up every 3 to 4 months was planned using available diagnostic tools (ultrasound, MRI, and hysteroscopy), since there are no immediate plans for pregnancy.

## Discussion

According to the 5th edition of the WHO Classification of Female Genital Tumours, APA is defined by an irregular, architecturally complex proliferation of endometrioid-type glands embedded within a myomatous or fibromatous stroma. Squamous morular metaplasia is a consistent histological feature. The glandular epithelium typically displays mild to moderate cytological atypia. The surrounding stroma is usually cellular yet cytologically benign, and is immunohistochemically negative for h-caldesmon, aiding in the distinction from true smooth muscle neoplasms.^6^ The observed pattern of nuclear β-catenin expression, stromal SATB2 reactivity, and CTNNB1 mutation provides a practical diagnostic clue, helping to distinguish these lesions from histological mimics, in line with previously published findings.^7^, ^8^, ^9^ This pattern underscores the contribution of Wnt/β-catenin pathway activation to the development and distinctive morphology of these tumors.^10^ Moreover, given the family history in the first case, MMR testing (MLH1, MSH2, MSH6, PMS2) is recommended to exclude Lynch syndrome, consistent with reported links between MMR defects and endometrial carcinogenesis.^11^ Complex architectural histopathological patterns observed in APA are frequently associated with concurrent abnormalities in the adjacent endometrial tissue (eg, endometrial hyperplasia without atypia, atypical hyperplasia, and endometrial carcinoma), and according to some authors, APA is “best regarded as analogous to a localized form of atypical endometrial hyperplasia.”^10^^,^^12^ Consequently, comprehensive surgical management should include excision of not only the lesion but also the surrounding endometrium and underlying myometrium, along with systematic random sampling of the endometrium to ensure thorough pathological assessment (eg, the four-step technique by Di Spiezio Sardo et al).^13^

A significant challenge in first patent was the rapid development of a new intracavitary tumor lesion detected just 3 months after the initial hysteroscopy, at which time the diagnosis of APA was established. This finding introduced novel diagnostic and therapeutic dilemmas. Ultrasound remains the primary diagnostic modality. Konstantinos et al have demonstrated that specific features identified on 3-dimensional power Doppler sonography can aid in differentiating APA from benign endometrial polyps and submucosal fibroids. Moreover, adenomyosis is present in more than two-thirds of APA cases.^14^ In our case, only a 2-dimensional transvaginal ultrasound was performed, revealing a heterogeneous lesion with hyperechoic areas but no direct or indirect signs of adenomyosis. However, histological examination during the repeated hysteroscopy confirmed the presence of adenomyosis, supporting the hypothesis by Longacre et al of a possible shared pathophysiological pathway. This mechanism may involve endometrial stromal progenitor cells capable of smooth muscle differentiation, potentially driven by prolonged estrogenic stimulation.^15^ Also, serum levels of CA 125 and CA 19-9 were assessed and found to be within normal limits in both patients. Although these tumor markers were not elevated, they may still serve as useful tools in the diagnostic evaluation and risk stratification of premalignant and malignant endometrial lesions.^3^

The risk of endometrial carcinoma in women with APA is reported to be approximately 8.8%, which is significantly higher than the overall risk of 0.8% observed in typical endometrial polyps.^16^ Hysterectomy remains the definitive treatment option; however, in women with fertility desire, fertility-sparing management remains a priority. The best outcomes have been reported with the four-step hysteroscopic technique described by Di Spiezio Sardo et al, which has since become widely used in the conservative management of APA. This approach has been associated with the lowest rates of disease recurrence and progression and is considered a valuable therapeutic option for patients with APA who wish to preserve their fertility.^13^ Mikos et al^17^ report that residual APA rates were significantly higher following dilation and curettage compared to all hysteroscopic approaches. A limitation of our report is the short follow-up period of 3 months, which precludes drawing firm conclusions regarding long-term recurrence or reproductive outcomes. However, no recurrence or progression was observed during follow-up; regular long-term monitoring is still recommended due to the potential risk of late recurrence. Also, patients should be counseled about the risk of recurrence, the potential for malignant transformation, and the likelihood of reduced fertility to ensure informed decision-making and appropriate follow-up planning.

Fertility-sparing treatment remains (Table) the greatest challenge, especially since most patients are nulliparous women in their thirties who desire future childbearing. Beshar et al,^18^ in a retrospective study reported that the highest rate of disease resolution was observed in patients treated hysteroscopically with intrauterine device placement, while the highest rate of progression to endometrial hyperplasia or carcinoma occurred in patients treated solely with hormonal progestogens. Interestingly, hysteroscopic resection alone appeared to be an effective and safe fertility-sparing treatment compared to hysteroscopic resection combined with hormonal therapy. Also, the potential advantage of hysteroscopic resection alone is the shorter duration of treatment, which allows patients to attempt conception earlier.^3^ By this approach, the first patient underwent exclusive surgical management due to her immediate desire for conception. Given the patient’s age, reduced ovarian reserve, and the risk of intrauterine adhesions after deep hysteroscopic resection, fertility potential is expected to be limited, highlighting the importance of appropriate counseling and individualized reproductive planning. Conversely, the second patient, who desired pregnancy but not at that moment, was treated with a combined modality involving surgical intervention and progestogen therapy via a levonorgestrel-releasing intrauterine device. Also, she was advised to plan pregnancy early to optimize fertility outcomes, emphasizing the importance of timely reproductive counseling in patients with potential fertility limitations.

**Table:** Fertility-sparing treatments for atypical polypoid adenomyoma with outcomes and follow-up

Treatment group	Study	No. of patients	Approach/method	Recurrence (rate/time)	Fertility outcome (pregnancy/live birth)	Follow-up (months)	Notes/limitations
**Hysteroscopic resection (TCR/piecemeal/4-step)**	**Di Spiezio Sardo et al**^13^	1	4-step TCR	0/1	Attempted spontaneous pregnancy	6	Case report; short follow-up
	**Dinas et al**^22^	1	Office hysteroscopy+D&C	0/1	NA	12	Case report
	**Yahata et al**^23^	1	3-step TCR	0/1	Pregnancy achieved; live birth	36	Case report
	**Matsumoto et al**^24^	10	D&C 9 cases, vaginal resection 2 cases, TCR 10 cases, hysterectomy 8	1/10	ND	39.6 (mean)	Retrospective cohort
	**Wang and Guo**^25^	44	4-step TCR	0/44	12 pregnancies (7 spontaneous, 5 via ovulation induction or ART)	42	Case series; APA-L/H subtypes
	**Domeniconi et al**^26^	1	TCR	0/1	Fertility preserved	ND	Case report
	**Wang et al**^4^	86	Hysteroscopic resection ± hormonal therapy	0 APA recurrence; 6 hyperplastic changes	Achieved pregnancies: 20/35; live births: 16/20	49	Single-center retrospective
	**Beshar et al**^18^	12	Hysteroscopic resection	∼33% progression/persistence	3 live births	54	Retrospective cohort
**Oral hormonal therapy (MPA, megestrol)**	**Nomura et al**^5^	18	High-dose MPA 3–9 mo	8/18 recurrences; 4 persistent	5 live births	22–142	Retrospective chart review
	**Chen et al**^27^	10	Progestin therapy	4/10 recurrences/persistence	7 pregnancies; 9 live births (3 IVF)	19–145	Retrospective cohort
	**Nomura et al**^5^	18	Maintenance hormonal therapy post-MPA	1/18 recurred after stopping maintenance	2 live births	22–179	Retrospective cohort
	**Tofoski et al**^28^	1	High-dose MPA	0/1	No conception	11	Prospective case series
**Combined therapy (hysteroscopic+hormonal/IUD)**	**Raffone et al**^3^	39	TCR+hormonal therapy (MPA/oral progestin)	12/39	Total 24/95 HT 4/18 TCR 8/38 TCR+HT 12/39	1–276	Systematic review
	**Casadio et al**^20^	11	Hysteroscopic resection+MPA	4/25 recurrence/progression	ND	39.2 ± 41.5	Retrospective multicenter
	**Beshar et al**^18^	12	Hysteroscopic resection+LNG-IUS/oral progesterone	∼33% progression/persistence	3 live births	54	Retrospective cohort
	**Solima et al**^29^	1	Hysteroscopic resection+LNG-IUS	1 recurrence pre-LNG	Pregnancy achieved; live birth	12	Case report
	**Narumi et al**^30^	1	Laparotomy resection+LNG-IUS	0	Pregnancy attempted after IUS removal	28	Case report
**LNG-IUS (alone or postresection)**	**Solima et al**^29^	1	LNG-IUS posthysteroscopy	0	Live birth	12	Case report
	**Narumi et al**^30^	1	LNG-IUS postlaparotomy	0	Pregnancy attempted after IUS removal	28	Case report

*ART*, Assisted Reproductive Technology; *D&C*, dilatation and curettage; *HT*, hormonal therapy; *IVF*, in vitro fertilization; *LNG-IUS*, levonorgestrel-releasing intrauterine system; *MPA*, medroxyprogesterone acetate; *NA*, not applicable; *NR*, not reported; *TCR*, transcervical hysteroscopic resection.

Currently, there are no official guidelines for fertility-sparing treatment in patients with APA. The most comparable recommendations are found in the ESGO/ESHRE/ESGE Guidelines for the fertility-sparing treatment of patients with endometrial carcinoma, which include suggestions for hysteroscopic management and hormonal therapy, megestrol acetate (160–320 mg/d) or medroxyprogesterone acetate (400–600 mg/d).^19^ On the other hand, Casadio et al suggest that hormonal therapy does not improve the outcomes of conservative treatment in APA. They speculate that the presence of fibromyomatous stroma may impair the responsiveness of APA to progestins.^20^ To date, there is a lack of data on pregnancy outcomes and success rates following the diagnosis of APA, given that fertility-sparing treatment is a relatively recent clinical approach. Additionally, more than one-third of patients are concurrently diagnosed with adenomyosis, an established independent risk factor associated with reduced reproductive outcomes.^21^

In conclusion, APA presents a notable diagnostic and therapeutic challenge, especially in women of reproductive age opting for fertility-sparing treatment. Given the risk of recurrence and progression, management should include complete hysteroscopic resection and close long-term follow-up to ensure early detection of potential malignant transformation.

## CRediT authorship contribution statement

**Ivo Vukasović:** Writing – review & editing, Methodology, Data curation, Conceptualization. **Dinka Pavičić Baldani:** Writing – review & editing, Visualization, Supervision. **Lana Škrgatić:** Writing – review & editing, Formal analysis. **Magdalena Karadža:** Writing – review & editing, Data curation, Conceptualization.
